# Pedestal High-Contrast Gratings for Biosensing

**DOI:** 10.3390/nano12101748

**Published:** 2022-05-20

**Authors:** Leonid Yu. Beliaev, Peter Groth Stounbjerg, Giovanni Finco, Ada-Ioana Bunea, Radu Malureanu, Lars René Lindvold, Osamu Takayama, Peter E. Andersen, Andrei V. Lavrinenko

**Affiliations:** 1 DTU Fotonik–Department of Photonics Engineering, Technical University of Denmark, Ørsteds Plads, Building 345A, DK-2800 Kongens Lyngby, Denmark; gfinco@phys.ethz.ch (G.F.); rmal@dtu.dk (R.M.); otak@fotonik.dtu.dk (O.T.); alav@fotonik.dtu.dk (A.V.L.); 2 DTU Health–Department of Health Technology, Technical University of Denmark, Ørsteds Plads, Building 345C, DK-2800 Kongens Lyngby, Denmark; s135331@student.dtu.dk (P.G.S.); lali@dtu.dk (L.R.L.); peta@dtu.dk (P.E.A.); 3 Optical Nanomaterial Group, Department of Physics, Institute for Quantum Electronics, ETH Zürich, Auguste-Piccard-Hof 1, HPT D5, 8093 Zürich, Switzerland; 4 DTU Nanolab–National Centre for Nano Fabrication and Characterization, Technical University of Denmark, Ørsteds Plads, Building 347, DK-2800 Kongens Lyngby, Denmark; adabu@dtu.dk

**Keywords:** refractometric sensing, high-contrast grating, silicon nanostructures, avidin, biotin, biosensing, bulk refractive index sensitivity, surface sensitivity, atomic layer deposition, nanofabrication

## Abstract

High-contrast gratings (HCG) are an excellent candidate for label-free detection of various kinds of biomarkers because they exhibit sharp and sensitive optical resonances. In this work, we experimentally show the performance of pedestal HCG (PHCG), which is significantly enhanced in comparison with that of conventional HCG. PCHGs were found to provide a 11.2% improvement in bulk refractive index sensitivity, from 482 nm/RIU for the conventional design to 536 nm/RIU. The observed resonance was narrower, resulting in a higher Q-factor and figure of merit. By depositing Al${}_{2}$O${}_{3}$, HfO${}_{2}$, and TiO${}_{2}$ of different thicknesses as model analyte layers, surface sensitivity values were estimated to be 10.5% better for PHCG. To evaluate the operation of the sensor in solution, avidin was employed as a model analyte. For avidin detection, the surface of the HCG was first silanized and subsequently functionalized with biotin, which is well known for its ability to bind selectively to avidin. A consistent red shift was observed with the addition of each of the functional layers, and the analysis of the spectral shift for various concentrations of avidin made it possible to calculate the limit of detection (*LoD*) and limit of quantification (*LoQ*) for the structures. PHCG showed a *LoD* of 2.1 ng/mL and *LoQ* of 85 ng/mL, significantly better than the values 3.2 ng/mL and 213 ng/mL respectively, obtained with the conventional HCG. These results demonstrate that the proposed PHCG have great potential for biosensing applications, particularly for detecting and quantifying low analyte concentrations.

## 1. Introduction

Refractometric sensing is an optical sensing scheme where a target analyte is detected by the change of refractive index in the vicinity of the sensing surface. This refractive index change occurs due to the presence of the analyte or due to changes in its concentration, which induce a measurable optical response as a read out. The key for high sensitivity in refractometric sensing is the presence of strong optical fields in the proximity of the sensor surface. Surface plasmon polaritons supported on metal films with negative permittivities have been extensively used for biochemical sensors [1,2,3] thanks to the tight localization of electromagnetic fields on the metal surface, which is favorable for the detection of an analyte. Plasmonic nanostructures that support highly localized plasmon resonances also have been studied for sensing applications, such as metal nanoparticles [4], gratings with one- [5] and two-dimensional periodicity [6], hyperbolic metamaterials [7,8,9], or other nanostructured metal surfaces [10]. An alternative approach with sensing platforms based on dielectric materials with positive permittivities has been developed. Transparent materials exhibit low absorption in the visible to near-infrared (IR) wavelengths, providing rather narrow resonances. Such dielectric sensing structures are exemplified by one- [11] and two-dimensional photonic crystals [12,13], Si particles [14,15,16,17], and metasurfaces [18,19,20].

Apart from the above-mentioned dielectric nanostructures, one-dimensional gratings, including high contrast gratings (HCGs), have been extensively studied due to their simplicity, narrow resonances and moderately high sensitivity [21,22,23,24,25,26,27,28,29,30,31]. A HCG consists of large refractive index grating bars surrounded by a low refractive index background. Typically, HCGs support guided-mode resonances [32] that propagate perpendicular to the grating bars with transverse magnetic (TM) polarization. Guided-mode resonances enable the detection of bulk refractive index changes of, e.g., water-glycerol mixtures [27], biotin-avidin binding [24], viruses [33,34], or cardiac biomarkers [35,36] for point-of-care (POC) applications. In order to realize the high refractive index contrast, Si is often used due to its high refractive index, low optical losses on the near-infrared wavelengths [35], and mature fabrication technology with mass production capability. Furthermore, the low absorptions of Si gratings do not produce any significant heating, which would change the local refractive index and result in measurement errors crucial for refractometric sensing.

**Figure 1:**
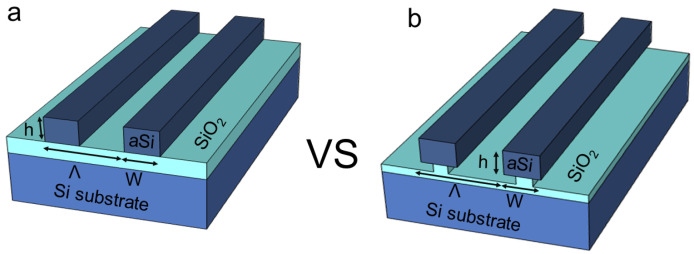
Schematic illustration cross-section of (**a**) conventional and (**b**) pedestal high-contrast gratings. The structural parameters of the gratings are defined by period Λ, grating height *h* and grating width *W*.

Here, we show that pedestal HCG structures (PHCG), shown in Figure 1, exhibit higher bulk and surface sensitivities than conventional HCGs. The PHCG offer a larger surface area for the analyte to interact with the electric fields of the guided-mode resonances supported in the grating [37,38,39], which enables larger resonance shifts in the presence of the analyte. Another advantage of the pedestal Si gratings lies in their even narrower resonance compared to conventional HCGs, which is advantageous for refractometric sensing. These two properties contribute to an overall enhanced performance of PHCGs over conventional gratings [30]. According to the numerical data in [30], the pedestal structure shows a 12% improvement in the bulk refractive index sensitivity. In order to demonstrate the improved sensitivity of the pedestal gratings, we characterized bulk refractive index sensitivity with mixtures of glycerol and deionized water. We conducted surface sensitivity measurements using dielectric layers with a thickness of a few nanometers as a model analyte. Finally, we measured the HCG surface sensitivity toward avidin after covalent functionalization with amino-propyltrimethoxysilane (APTMS) and biotin. We found that the bulk sensitivity of the pedestal gratings is 536 nm/RIU (refractive index unit) as opposed to 482 nm/RIU for the conventional ones. PHCGs also show over 10% improvement in surface sensitivity, as evaluated after the atomic layer deposition (ALD) of oxide layers with different thicknesses and different refractive indices. The limit of detection (*LoD*) for avidin was estimated as 2.1 ng/mL for PHCG and 3.2 ng/mL for the conventional one, while the limit of quantification (*LoQ*) is 85 ng/mL for the pedestal and 213 ng/mL for the conventional HCG. Thus, the overall sensing performance of PHCG outperforms that of the conventional HCG confirming its suitability for biosensing applications.

## 2. Experimental Section

**Figure 2:**
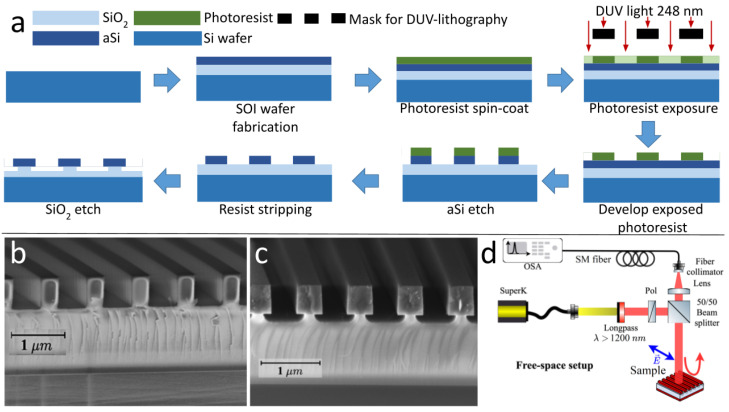
(**a**) Schematic of the fabrication process for the pedestal-grating, (**b**) SEM image of the HCG in cross-section, (**c**) SEM image of the PHCG in cross-section, (**d**) the optical setup.

**Fabrication**. All the work was carried out in a class 10–100 cleanroom facility. The main steps of the fabrication process are shown in Figure 2a. The 500 μm thick Si ⟨100⟩ wafers went through a standard RCA cleaning procedure. The wafers were oxidized in a conventional quartz tube (furnace from Tempress) using a wet oxidation process based on H_2_O at 1100 ${}^{\circ }$C, resulting in a 1.1 μm SiO${}_{2}$ layer on Si. Next, a 500 nm thick amorphous Si (aSi) layer was deposited on top by a low pressure chemical vapor deposition (LPCVD) (furnace from Tempress) based on SiH${}_{4}$ (silane) at 560 ${}^{\circ }$C. This procedure enables the preparation of home-made silicon-on-insulator (SOI) substrates. The thicknesses of the SiO${}_{2}$ layer on Si and aSi on top were chosen basen on theoretical calculations [30].

A one-dimensional periodic lattice of bars was then patterned onto the Si surface by conventional deep-UV lithography (DUV stepper: Canon FPA-3000 EX4, Canon, Blackwood, NJ, USA). The lattice parameters are the following: lattice period Λ= 820 nm and bars width w= 340 nm. The procedure includes the steps: coating and baking of a 65 nm bottom anti-reflective coating layer (BARC) and 360 nm positive photoresist KRF M230Y, exposed (exposure dose of 240 J/m${}^{2}$) on a patch with sizes of 0.5 × 0.5 cm${}^{2}$ and post-development. Thereafter, deep reactive ion etching (DRIE, DRIE Pegasus from SPTS, SPTS Technologies Ltd., Newport, UK) was performed to etch down bars through the Si layer. The processing temperature was kept at 0 ${}^{\circ }$C and the process pressure at 10 mTorr. The process consists of three steps: the BARC etch using O${}_{2}$ plasma, SF${}_{6}$ with accurate nanoscale directional etching of silicon and resist removal using O${}_{2}$ plasma [40]. In order to realize the pedestal HCGs, the HF vapor phase (Primaxx uEtch from SPTS) is used for the controllable etching of silicon dioxide. The process includes pressure and temperature stabilization in the presence of N${}_{2}$ (1425 sccm) and (EtOH) 210 sccm gasses; the etch step, when HF 190 sccm flow is introduced into the chamber and the pumping. The etch time was 600 s. The shape of the produced structures was investigated in top and cross-sectional view by scanning electron microscopy (SEM, Zeiss, Jena, Germany), as shown in Figure 2b,c.

For the surface sensitivity measurements, the structures were coated with 2–5 nm thick layers of Al${}_{2}$O${}_{3}$, HfO${}_{2}$ and TiO${}_{2}$ using ALD. The ALD of oxides is based on two types of precursors—H${}_{2}$O as the oxidation agent and trimethylaluminum (TMA), titanium tetrachloride (TiCl${}_{4}$) or tetrakis (ethylmethylamido) hafnium (TEMAHf) as sources of aluminum, titanium and hafnium, respectively. The deposition process was carried out at 200 ${}^{\circ }$C, 150 ${}^{\circ }$C and 350 ${}^{\circ }$C and the deposition rates were 0.097 nm/cycle, 0.0385 nm/cycle and 0.090 nm/cycle, correspondingly. Such thicknesses are difficult to see by using cross-sectional (SEM) measurements, but ellipsometry and X-ray reflectivity spectroscopy (XRR, Rigaku, Austin, TX, USA) performed on pieces of silicon substrates confirmed the deposited thicknesses [41].

**Figure 3:**
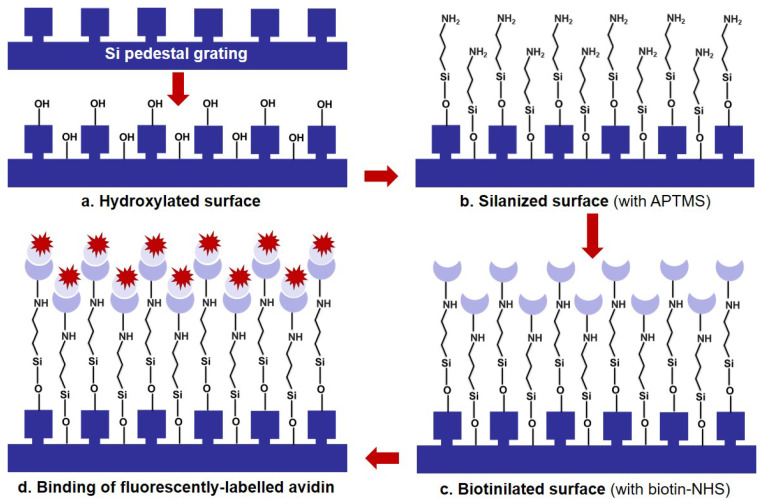
Functionalization protocol for the optical biosensor. (**a**) The Si pedestal grating is hydroxylated by piranha treatment, (**b**) Silanization of the surface results in an APTMS monolayer, (**c**) The surface is biotinilated using a biotin-NHS compound that covalently bonds to the amino groups of APTMS, (**d**) The fluorescently-labelled avidin is recognized by the biotin and thus bound to the surface.

**Functionalization**. The functionalization protocol is schematically shown in Figure 3. In the first step (Figure 3a) the samples were placed in a piranha solution (sulfuric acid and hydrogen peroxide in 4:1 ratio) for one hour. This removes any potential organic contaminants and ensures the presence of hydroxyl groups on the surface. In the second step (Figure 3b) the samples were placed in a container with a 3 mM solution of APTMS in anhydrous toluene (150 mL toluene and 75 μL aminosilane) and allowed to incubate at room temperature for 30 min in a laminar flow bench. After this step, the samples were rinsed with toluene (2×) and methanol (2×) and blow-dried with nitrogen. The silanization step was optimized in-house based on the original protocol from [42], with the main difference being that we employ a reduced silane incubation time. Subsequent functionalization steps were performed according to the Sigma Aldrich (Merck KGaA, Darmstadt, Germany) protocol provided together with the chemical compounds. Briefly, in the third step (Figure 3c), the biotin-NHS compound was dissolved in PBS to a concentration of 10 mM. 75 μL of the solution were added to each sample and allowed to react for 30 min. In the fourth and final step (Figure 3d), the fluorescently-labelled avidin derivative avidin-sulforhodamine 101 was dissolved in PBS at concentrations ranging from 71 pg/mL to 7.1 μg/mL. 75 μL of the solution were added to each sample and allowed to react for 30 min. Reference samples were prepared using the same protocol, except that the second step (silanization) was skipped.

**Optical characterization**. Free-space reflectance measurements with a resolution of 0.1 nm were performed with the normally incident TM-polarized light (Figure 2d). The light source was a supercontinuum broadband laser (SuperK; NKT Photonics A/S, Birkerød, Denmark). For the reference spectrum, the TM reflectance from a gold mirror was taken. The presented reflectance is the average of 10 scans. The signal was guided to the optical spectrum analyser (OSA, ANDO AQ-6315E, Yokogawa, Tokyo, Japan) by means of a single mode fiber. To avoid additional heating of the structure, a longpass-filter at 1200 nm was used.

## 3. Results and Discussion

**Bulk refractive index sensitivity measurements**. In order to evaluate the performance of the HCG sensors, the bulk refractive index sensitivity, $S_{B}$, is defined as

(1) $$S_{B}=\frac{\Delta \lambda }{\Delta n}\left[\mathrm{nm}/\mathrm{RIU}\right],$$

where Δλ is the shift of the resonance wavelengths as opposed to the change of background (bulk) refractive index Δn, and RIU stands for the refractive index unit. Note that the bulk refractive index sensitivity does not always coincides with the surface sensitivity for nanometer-thick analyte layers on the surface [30]. The overall performance of sensing structures is characterized by figure of merit (*FOM*), which relates the wavelength shift, Δλ, or bulk refractive index sensitivity, $S_{B}$, and the quality of resonance or the full width at half maximum (*FWHM*):

(2) $$FOM=\frac{S_{B}}{FWHM}.$$

**Figure 4:**
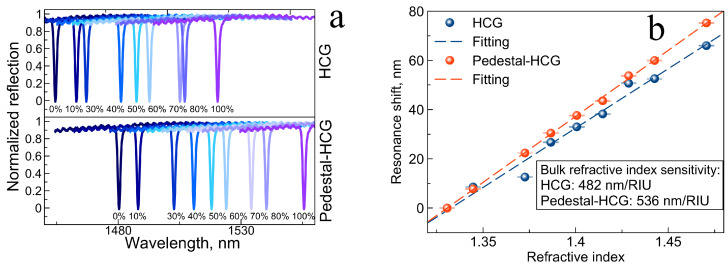
(**a**) Reflectance spectra of the HCG (top part) and the pedestal HCG (bottom part) in glycerol-aqueous solutions, (**b**) resonance wavelength of the HCG (blue) and PHCG (red) versus the change in RI associated with different glycerol concentration in the solution.

In order to determine $S_{B}$, we immersed the sensor in solution with glycerol diluted in deionized (DI) water (0 to 100% *v*/*v*), which corresponds to the RI variations from 1.33 to 1.47 RIU [8,27,43]. These values are also chosen to mimic a dielectric environment relevant to label-free assays. The change in RI results in a red-shift of the resonance, as shown in Figure 4a,b. The response curves are linear and allowed to calculate a sensitivity of 482 nm/RIU for the conventional HCG, which is higher than corresponding sensitivities reported for other HCG sensor devices [35]. For the PHCG, the calculated $S_{B}$ is 536 nm/RIU, 11.2% higher than for the conventional HCG (Figure 4b). Moreover, the dip for resonant reflections has a FWMH of 1.1 and 0.95 nm for the HCG and PHCG, respectively. This is more than two times narrower than the values measured in air, which were 2.61 and 2.45 nm, respectively. Calculated FOM in the solutions were 438 RIU${}^{-1}$ and 564 RIU${}^{-1}$, correspondingly, with Q-factors of 1.3 × 10${}^{3}$ and 1.5 × 10${}^{3}$.

**Figure 5:**
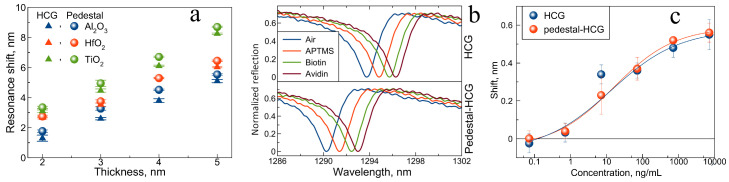
(**a**) Position of the resonance for the HCG (triangles) and the pedestal-HCG (spheres) as a function of deposited thicknesses of Al${}_{2}$O${}_{3}$ (blue), HfO${}_{2}$ (red) and TiO${}_{2}$ (green), (**b**) reflectance spectra of the HCG (top part) and the PHCG structure (bottom part) after each of the functionalization steps, (**c**) measured resonance shifts for avidin molecule detection in PBS for the conventional (blue) and pedestal (red) HCGs.

**Surface sensitivity measurements**. To evaluate the surface sensitivity of our devices, layers of 2 to 5 nm thick aluminum, hafnium, and titanium oxides with refractive indices of 1.6, 1.9, and 2.4, respectively, were deposited on the surface of the HCGs. The results of the measurements are shown in Figure 5a. It can be seen that an increase in the thickness of the deposited film leads to a red shift of the dip in the reflection spectrum. Additionally, this shift is higher for oxides with a higher refractive index. Figure 5a shows a comparison of shifts for the HCG and the PHCG structures. The performance of the pedestal structure is 10.5% better than that of the conventional structure, which is associated with the larger effective sensing surface on PHCG [37,38].

**Protein detection**. The limit of detection (*LoD*) and limit of quantification (*LoQ*) are two important performance characteristics in sensor validation [44,45]. *LoD* is defined as the the lowest concentration of an analyte that can be detected, while *LoQ* is the lowest concentration of an analyte that can be quantified with acceptable precision and accuracy [44,45]. They can be expressed as:

(3) $$LoD=blank+\frac{3SD}{S}$$

(4) $$LoQ=blank+\frac{10SD}{S},$$

where is *S* is the slope of the calibration curve and SD is the standard deviation of the blank.

In order to validate the device as a potential biosensor, the resonance shift, Δλ, was measured in air after each functionalization step, i.e., piranha cleaning, coating with APTMS, adding of biotin as an antibody model and avidin as an antigen model. The last two compounds were chosen as a model of antibody-antigen interactions due to their well-known strong binding mechanism and relatively low cost [46]. Additionally, the molecular weight of avidin of around 66 kDa is comparable to that of many proteins and biomarkers. A sequential red-shift was observed for each of the functionalization stages, as shown in Figure 5b. For quantitative measurements of avidin, the position of the dip in the reflectance spectrum after the addition of biotin was chosen as a blank. Then, six different concentrations of avidin between 71 pg/mL and 7.1 μg/mL were added to the structures. Three independent measurements were taken for each concentration. The obtained detection curve for both types structures is shown in Figure 5c. The error bars represent the standard deviation of three independent measurements. The response curves exhibited a standard sigmoidal-shape and were fitted by the Logistic function [47,48]:

(5) $$\Delta \lambda =A_{1}-\frac{A_{2}}{1+{\left(C/A_{3}\right)}^{A_{4}}},$$

where *C* is the concentration of the analyte, $A_{1}$, $A_{2}$, $A_{3}$ and $A_{4}$—fitted constants that were equal to 0.58548, 0.6989, 9.90319, 0.41903 and 0.58919, 0.63468, 15.87538, 0.51237 for the HCG and PHCG, respectively. The values $R^{2}$ for PHCG and HCG were 0.98656 and 0.94874, respectively. As a result, the estimated *LoD* is 3.2 ng/mL for the HCG and 2.1 ng/mL for PHCG. The *LoQ* is 213 ng/mL and 85 ng/mL, respectively. The observed data show pronounced improvement in both *LoD* and *LoQ* values of PHCG compared to the conventional one.

To verify that the functionalization proceeds according to the protocol, experiments were carried out to measure the spectral shift for the biotin/avidin system added on the surface of the device in the absence of APTMS. The shift measured for the reference samples was about 0.24–0.27 nm, significantly smaller than that measured for the samples prepared using the full functionalization protocol. This confirms the role of the APTMS layer as cross-linker in the system and clearly shows the advantage of covalent biotin/avidin immobilization on the silanized surface, in contrast to simply relying on protein adsorption on the clean sample surface.

Finally, the comparison of overall performance between the two types of HCG structures is summed up in Table 1.

## 4. Conclusions

To conclude, we characterized the sensing performance of pedestal and conventional HCGs. With the increased surface area that can interact with the analyte, we anticipated that the PHCG should outperform the conventional one. Indeed, we observed an improvement of 11.2% (536 nm/RIU against 482 nm/RIU) in bulk sensitivity and a 10.5% improvement in surface sensitivity measured with different oxide layers deposited by ALD. These results are in full agreement with the modeling of similar structures, where a 12% improvement in surface sensitivity was shown [30]. Such remarkable quantitative correlation between numerical (ideal) and measured results certifies the quality of samples and the accurateness of the experimental routines. Furthermore, surface sensitivity toward a model analyte in solution, avidin, was measured after HCG covalent functionalization with APTMS and biotin, demonstrating a significant improvement in *LoD* (2.1 ng/mL as opposed to 3.2 ng/mL) and *LoQ* (85 ng/mL and 213 ng/mL, respectively) for the PHCG samples. Thus, we conclude that the pedestal HCG as a biosensor platform has superior potential for the detection of various kinds of analytes in comparison with the conventional HCGs.

## Figures and Tables

**Table 1:** Summary of the measured sensing performance of conventional and pedestal HCGs.

HCG	Quality	Figure of	Bulk Refractive	Limit of	Limit of
Sensing	Factor	Merit	Index Sensitivity	Detection	Quantification
Platform		(*FOM*)		(*LoD*)	(*LoQ*)
		[RIU${}^{-1}$]	[nm/RIU]	[ng/mL]	[ng/mL]
Conventional	(1.3 ± 0.1) × 10${}^{3}$	438 ± 2	482 ± 2	3.2 ± 0.1	213 ± 11
Pedestal	(1.5 ± 0.1) × 10${}^{3}$	564 ± 2	536 ± 2	2.1 ± 0.1	85 ± 13

## Data Availability

The data presented in this study are available on request from the corresponding author.

## Abbreviations

The following abbreviations are used in this manuscript:

HCG	High-contrast grating
PHCG	Pedestal high-contrast grating
RIU	Refractive index unit
*LoD*	Limit of detection
*LoQ*	Limit of quantification
POC	Point-of-care
APTMS	Amino-propyltrimethoxysilane
ALD	Atomic layer deposition
SOI	Silicon-on-insulator
TMA	trimethylaluminum
TEMAHf	Tetrakis (ethylmethylamido) hafnium
SEM	Scanning electron microscopy
XRR	X-ray reflectivity spectroscopy
*FOM*	Figure of merit
*FWHM*	Full width at half maximum
